# Habitat Temperatures of the Red Firebug, *Pyrrhocoris apterus*: The Value of Small-Scale Climate Data Measurement

**DOI:** 10.3390/insects14110843

**Published:** 2023-10-30

**Authors:** Helmut Käfer, Helmut Kovac, Anton Stabentheiner

**Affiliations:** Institute of Biology, University of Graz, 8010 Graz, Austria; anton.stabentheiner@uni-graz.at

**Keywords:** *Pyrrhocoris apterus*, microclimate, critical thermal limits, habitat temperatures

## Abstract

**Simple Summary:**

Ambient temperature is a main external parameter in the life of ectothermic insects. It affects egg and larval development as well as adults’ survival, thriving and propagation, and successful overwintering. We conducted temperature measurements in Central Europe in the habitat and in the microhabitats of *Pyrrhocoris apterus*, a herbivorous bug species almost ubiquitous in Eurasia, with a high invasive potential (USA, Central America, India and Australia). and set them against freely available climate data commonly used to characterize habitat climate. Our temperature measurements were also compared to the bug species’ thermal limits (critical thermal minima and maxima). Ambient temperatures outside the thermal boundaries of *P. apterus* can and do occur in the habitat. Microhabitat measurement showed that in summer, individuals simply moved from hot areas to cooler ones, and in winter they sought areas with bearable temperatures for hibernation. Temperatures in these particular areas are not (always) represented in large-scale climate tables, leading to possible misinterpretation of the future dispersal behavior, e.g., of invasive species.

**Abstract:**

Ambient temperature is a main parameter that determines the thriving and propagation of ectothermic insects. It affects egg and larval development as well as adults’ survival and successful overwintering. *Pyrrhocoris apterus* is a herbivorous bug species almost ubiquitous in Eurasia. Its distribution extends from the Atlantic Coast to Siberia, Northwest China and Mongolia. After introduction, it established successfully in the USA, Central America, India and Australia, which indicates a high invasive potential of this species. We determined the climatic conditions in Central Europe in a habitat where *P. apterus* has been continuously observed for decades. We conducted temperature measurements in the habitat and in the microhabitats where individuals could be found during the year and set them against freely available climate data commonly used to characterize habitat climate. Our temperature measurements were also compared to thermal limits (critical thermal minima and maxima). Although ambient temperatures outside the thermal boundaries of *P. apterus* can and do occur in the habitat, the bugs thrive and propagate. Microhabitat measurement in winter showed that individuals sought areas with favorable temperatures for hibernation. In particular, these areas are not (always) represented in large-scale climate tables, leading to possible misinterpretation of future patterns of spread of invasive species spread.

## 1. Introduction

The Pyrrhocoridae is a family of phytophagous Heteroptera, or true bugs, predominantly distributed in the Paleotropical or subtropical Palearctic [1]. The red firebug (*Pyrrhochoris apterus*) is one of the few species that expanded its range to the temperate zone of the Palearctic. It is now an almost ubiquitous species widely distributed from the west of Europe to the south of western Siberia and Northwest China [2,3]. Recently, it has been spreading eastward to the Mediterranean area in Europe [4] and northward where it has reached Norway [5]. It has also been reported from the US, Central America, and India [1] and also Australia [6]. This ability to spread and establish viable populations in differing habitats shows a considerable ability to cope with a broad variety of environmental factors.

Temperature is a major factor limiting the spread and thriving of ectothermic insects [7,8,9]. It determines physiological processes in the development of eggs, larvae and adults, and thus their survival and reproductive success. Once temperatures are outside a species’ favorable or at least viable range (limited by the critical thermal minimum and maximum), they become detrimental or, at some point, lethal [10]. In Central Europe, the change in ambient temperatures regarding the species’ thermal borders is a seasonal occurrence, with low winter temperatures and (possible) higher temperatures in summer. *P. apterus* copes with the respective adverse conditions by hibernating in sheltered spots and by seeking out cooler, more favorable parts of the habitat in the short term.

**Figure 1:**
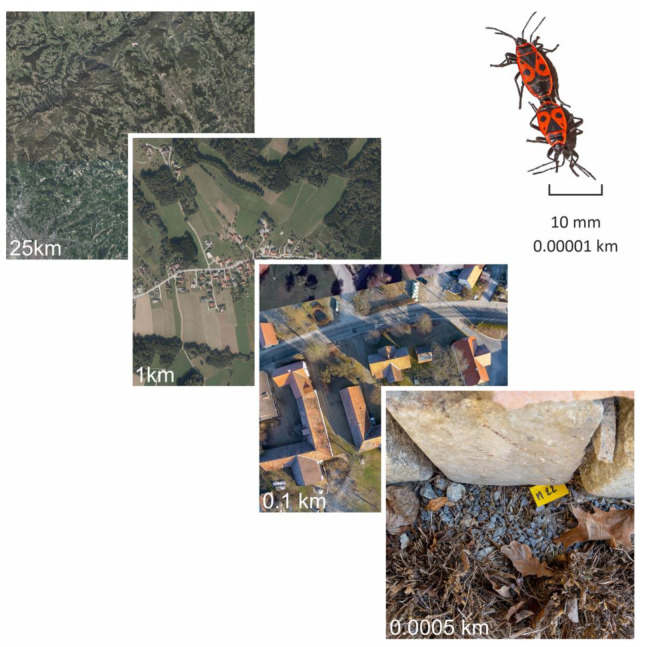
Area sizes usually used for climate data measurement compared to the size of the (micro) habitats of local organisms with the respective image square’s side length in kilometers. A size ratio of 10,000:1 in terms of measurement area:organism size can easily occur in ecophysiological research. The 25 km and 1 km overview shots are courtesy of Land Steiermark//GIS Steiermark 2021.

Previous attempts were aimed at modelling the distribution of ectotherms and endotherms in relation to macroecological climate parameters and latitudes, as well as thermal tolerance [11,12,13,14,15,16,17]. Contradicting hypotheses on the impact of climate warming on ectotherms (see [18] versus [19]) indicate inherent shortcomings of large-scale climate-based studies describing biological effects. The discrepancy in scale of climate measurements to the respective animals (climate data point: animal size = appr. 10,000-fold [20]) is certainly a main problem (Figure 1). A habitat’s microclimate can act as a buffer for large-scale environmental conditions. Recently, more and more studies have taken microclimate data into account [21,22,23,24,25] in order to model the external conditions for organisms under changing environmental conditions more accurately. However, even microclimate measurements do not always show the whole picture, as the animals are not stationary. In reaction to uncomfortable conditions, the animals usually have a repertoire of physiological (e.g., evaporation [26,27,28]) or behavioral responses (e.g., burrowing into the ground, or simply relocating [29,30]).

In this study, we recorded small-scale temperature data from an established *Pyrrhocoris apterus* habitat in Central Europe and compared these with freely available climate data from national and international sources in order to show whether such large-scale data are an appropriate measure for characterizing a species’ habitat conditions. We hypothesize that temporally and spatially coarse-resolution temperature data do not accurately reflect immediate conditions in the (micro)habitat of *P. apterus*. We discuss the influence of large- and small-scale measurements in terms of predicting thriving and dispersal in the context of the thermal limits of the species.

## 2. Materials and Methods

### 2.1. Animals and Habitat

We measured habitat temperatures in rural Gschwendt (47.17855° N, 15.5729° E, 521 m ASL, Styria, Austria, Central Europe) in the yard of an old farmhouse (Figure 1) throughout the years 2014 to 2016, where a stable population of *Pyrrhocoris apterus* could be observed for more than 20 years.

### 2.2. Temperature Measurement and Climate Data

**Figure 2:**
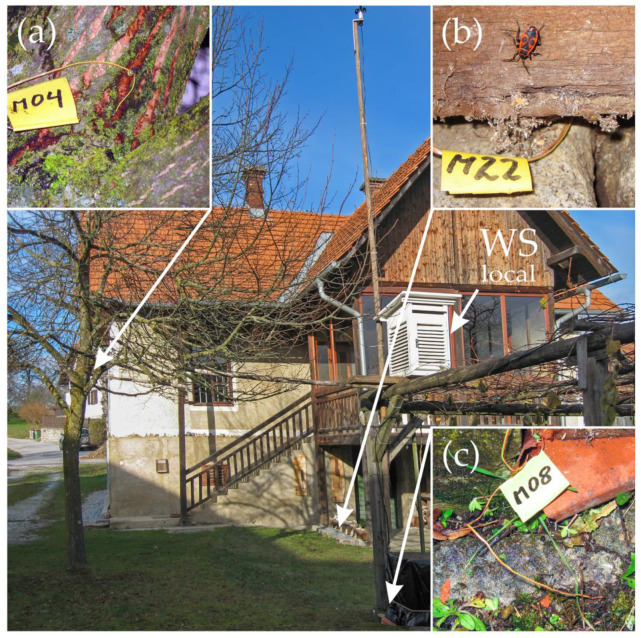
Area and habitat where temperature data were measured for *P. apterus*. The inserts show detailed positioning of the thermocouples (**a**) at a branch in the crown of a linden tree, (**b**) in a crevice between cobblestones at the farmhouse’s porch and (**c**) at the post of the vine trellis near the ground. Notice the standardized weather station (WS_local_, white box right of center in the main picture) providing on-site weather data.

Local weather/climate data such as air temperature, relative humidity and wind speed were sampled with a weather station according to the standard meteorological collection of climate data (Figure 1, WS_local_), positioned directly at the sample site. For measurement of the bugs’ microhabitat temperatures, NiCr/Ni thermocouples were placed at relevant points where individuals could be regularly observed at different times of the year (spring and summer common areas, winter hibernaculum), i.e., on a linden tree (*Tilia* sp.) at the ground near the roots, at the trunk’s bark, and at a branch in the crown (Figure 2a), in crevices in the ground between cobblestones by the porch of the main building (Figure 2b), and on the ground near a vine (*Vitis vinifera*) trellis stand (Figure 2c). The data were recorded with a data logger (ALMEMO 5590-2, Ahlborn GmbH, Holzkirchen, Germany) at 10 min intervals.

Standard temperature data were also provided by the ZAMG (Central Institute for Meteorology and Geodynamics, now Geosphere Austria, Vienna, Austria) from the official weather station closest to the sample site (Gleisdorf, 15.708055° E 47.115555° N, 377 m ASL, appr. 20 km distance; monthly and daily minimal, maximal, mean temperatures). All large-scale climate data were obtained by ZAMG’s homogenized network of weather stations in Austria (SPARTACUS: calculated daily and monthly mean temperatures, 1 × 1 km grid; INCA_L: 1 × 1 km grid, hourly resolution, ensemble data) via the ZAMG data hub [31]), as well as from ECA&D’s E-OBS dataset (calculated daily min, max, mean temperatures, 0.25 deg raster [32,33,34]). For details on the type of processed data, see Table 1. Where applicable, all temperature data were calculated from the higher to the lower temporal resolution. An interpolation in the opposite direction was not made.

The study site was located in the warm temperate zone (Köppen–Geiger Climate Classification zone Cfb: warm temperate fully humid with warm summer [35,36,37]).

### 2.3. Thermal Limits and Safety Margins

Thermal limits were assessed based on Käfer et al. [38,39], following established methods [40,41,42,43,44,45,46]. The method used takes the so-called “knockdown point” (i.e., cease of coordinated movement) as an indication for the critical thermal minimum (CT_min_) and maximum (CT_max_). Positive differences of CT_min_ or CT_max_ to the measured minimum and maximum temperatures the animals would be exposed to in this habitat are interpreted as a low risk of temperature stress and performance decrease from low and high temperatures (see [13,47]).

### 2.4. Comparison of Measured and Provided Temperature Data

We compared large-scale temperature data from publicly available sources (ZAMG, ECA&D, Table 1) with our own small- and microscale measurements (see Table 2). Differences in the various time bases of the calculations or observations were adjusted, whereby calculations were always made from the higher to the lower resolution (data reduction of higher resolutions, e.g., 10 min to 1-day intervals), not in the opposite direction (interpolation from datasets with a coarser temporal resolution). Due to partially missing measured data (technical problems with data recording: water intrusion in 2014, a lightning strike in 2016; see Supplementary Materials S1), the comparison of averaged weekly and monthly temperatures was performed only for 2015.

**Table 2:** Designation and location of temperature sensors (thermocouples) measuring ambient air temperature, as well as designation of relevance for a season (spring, summer, autumn, winter). Sensors and locations in bold indicate winter hibernacula. For details on positioning, see Figure 2 and Section 2.2. AGL = above ground level.

Sensor #	Location	Season
M00	WS_local_; weather station on site, 2.7 m AGL (above ground level)	s, s, a, w
**M02**	***Tilia* sp. near the roots, north (shade), 1 cm AGL; near winter hibernaculum**	**s, s, a, w**
M03	*Tilia* sp. trunk; north (shade), 100 cm AGL	s, s, a
M04	*Tilia* sp. crown, 250 cm AGL	s, s, a
M05	Trellis post; south (sun), 1 cm AGL	s, s, a
M06	Trellis post; north (shade), 1 cm AGL	s, s, a
**M07**	**Trellis post; north (shade), soil, under fall foliage; near winter hibernaculum**	**s, s, a, w**
M08	Trellis post; south (sun), 1 cm AGL	s, s, a
**M22**	**Porch, crevices between cobblestones; winter hibernaculum, −10 cm AGL**	s, s, **a, w**

## 3. Results

**Figure 3:**
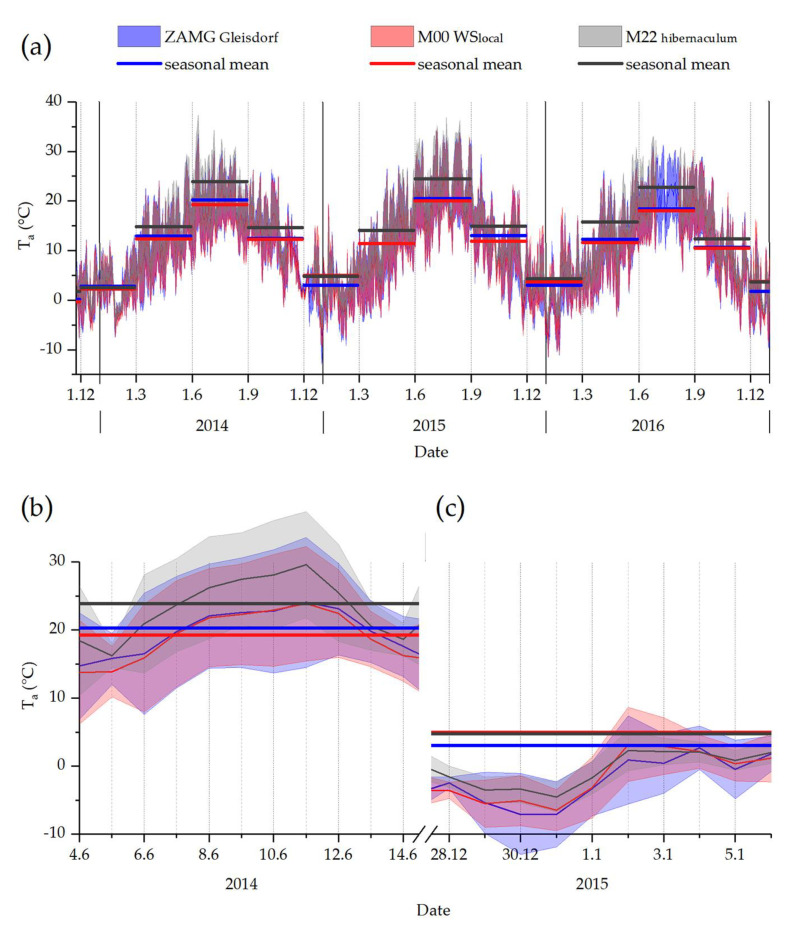
Freely available daily temperature data from the ZAMG Gleisdorf weather station (blue; daily minima/maxima) as well as actual temperatures measured locally at the study site with a standardized weather station (M00 WSlocal, red; WS_local_ in Figure 2), and inside a firebug winter hibernaculum (M22 hibernaculum, grey); (**a**) shows the entire observation period, (**b**) shows the warmest days in summer 2014, (**c**) the coldest days in winter 2014–15; daily minima/maxima were calculated from 10 min interval data. Seasonal mean temperatures were calculated for ZAMG Gleisdorf and our local weather station data (blue and red horizontal lines), as well as for the firebug winter hibernaculum (M22; dark grey horizontal lines).

Measured (raw) data and processed gridded data differed, sometimes considerably, depending on time and measurement location. Table 3 shows temperatures physiologically relevant for the bugs, as there are the absolute minimum temperatures in winter seasons (relevant regarding CT_min_) and absolute maximum temperatures in summer seasons (relevant for survival at CT_max_ temperatures) throughout the entire experimental period. Our own standardized local weather station (M00) served as the basis for the temperature at our study location. Differences from this “baseline” are listed for external data sources (ZAMG, ECA&D) and for our own measurement points in the microhabitat, relevant for the winter (i.e., cold) and summer (i.e., hot) seasons. Positive deviation from the baseline indicates higher temperatures at the respective sample points (i.e., the baseline underestimates the temperatures at this sample point), and negative deviation indicates the opposite (i.e., overestimation of the temperature at the particular measurement points by the baseline). Exceptionally high positive deviation values for certain thermocouples are caused by direct sunlight on the sensor. This would resemble bug temperature if they were in that position for a longer period. In winter (1.12. to 1.3.), the ZAMG Gleisdorf data always underestimated the temperatures measured by the local weather station (M00) at our study site: 2013–14: −0.1 °C, 2014–15: −3.5 °C, 2015–16: −0.1 °C. However, the minimum temperatures measured at the firebugs’ winter hibernaculum (M22) were always significantly higher than measured by ZAMG Gleisdorf (2013–14: +1.6 °C; 2014–15: +6.3 °C; 2015–16: +5.0 °C) and also M00 (2013–14: +1.5 °C; 2014–15: +2.8 °C; 2015–16: +4.9 °C; see Table 3, Table S1). Figure 3 shows daily (min and max) and seasonal (calculated means) temperature data from the nearest official weather station (ZAMG Gleisdorf), a standardized weather station on site (M00), and exemplary data from a microclimate sample point underground (M22, winter hibernaculum).

Table 4 shows averaged monthly temperature data for 2015 for all monitoring points in the habitat, as well as the provided external data (see Table S2 for weekly averages). In winter, all averages never fell below 0.9 °C, while the absolute values did (see Table 3). In summer, the mean monthly temperatures never reached values that would have been critical for the species. There were no significant differences, neither between measured and calculated data nor within data groups (multiple average comparisons, 95%, LSD).

**Figure 4:**
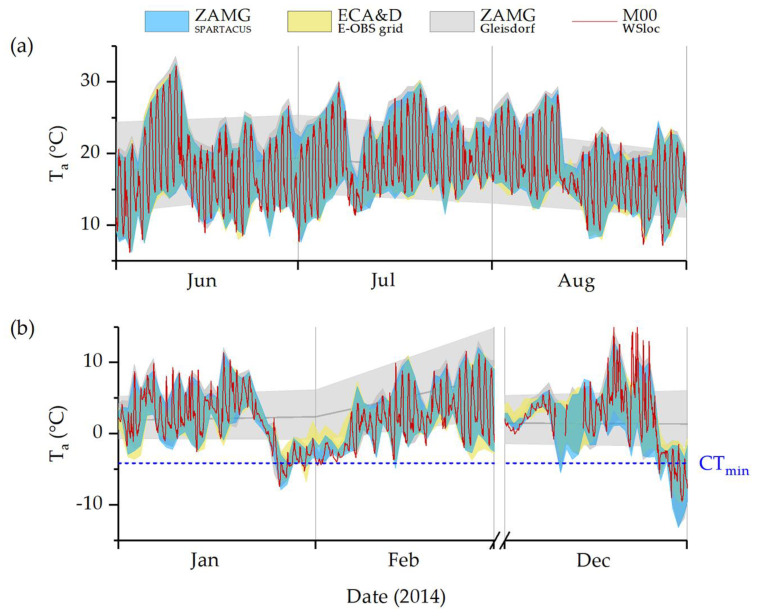
Freely available interpolated temperature data from ECA&D (yellow, daily minima/maxima, gridded data, worldwide) and ZAMG (grey, monthly means and minima/maxima from one station; blue, daily minima/maxima from gridded data, Austria), as well as actual temperatures measured at 10 min intervals at the sample site with a standardized weather station (M00 WSloc, red line) for 2014 (**a**) summer and (**b**) winter seasons. CT_min_ data from [39].

Figure 4 shows temperature estimations from ZAMG and ECA&D for the habitat as well as temperatures measured with our weather station during the winter and summer months (meteorological seasons) of 2014. While the daily resolution estimates (blue, yellow) reflected the measured temperatures (red line) at the study site reasonably well, the monthly averages (grey) from the measuring station nearest to the sample habitat (ZAMG Gleisdorf) did not represent the temperatures relevant to *P. apterus* well. Especially, diurnal temperature extremes sometimes differed considerably. The daily estimates seem to be more accurate in summer, especially regarding minimum and maximum air temperatures (see also Table 3).

**Figure 5:**
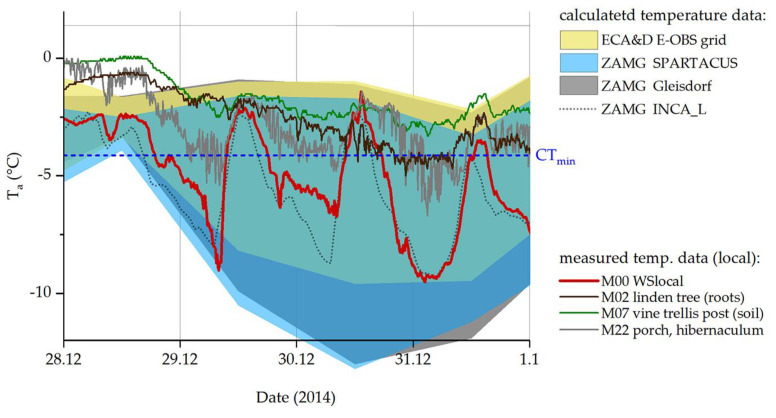
Temperatures on the coldest days of winter 2014. ZAMG (dark grey and blue areas) and ECA&D (yellow area) daily maxima and minima. The black dotted line shows calculated hourly data (ZAMG, INCA_L), the red line shows our measured temperature at 10 min intervals (M00, weather station on-site). Temperatures measured at the sample site are represented by colored lines (M02: linden tree, roots; M07: vine trellis; M22_Hiber_: cobblestones; all near or directly in winter hibernacula of *P. apterus*). CT_min_ from [39].

Figure 5 shows the discrepancies between calculated and measured-on-site temperature data during the coldest winter days of 2014. Monthly means of the nearest ZAMG weather station (grey area) keep well above the insects’ CT_min_ at all times. Daily estimates of minimum and maximum temperatures based on large-scale grids (ZAMG and ECA&D; blue and yellow areas) match our own, local temperature measurements better. However, even our data measured at 2.7 m above ground (red line; M00 WSloc) do not depict the conditions in the winter hibernacula (M22) and on the ground (M02, M07) accurately, where temperatures rarely fall below the species’ CT_min_.

**Figure 6:**
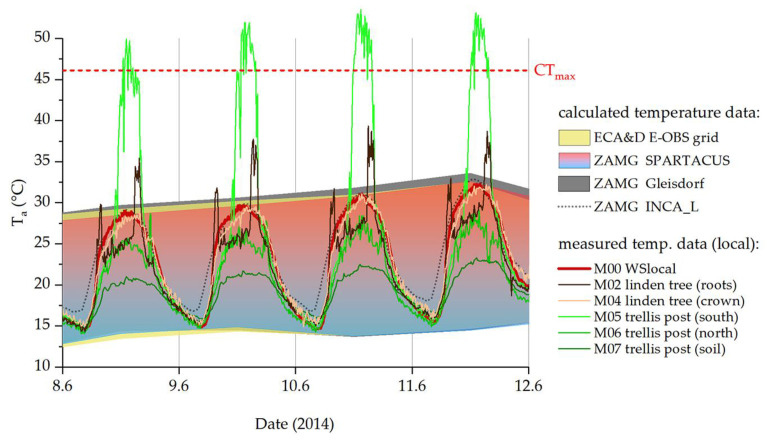
Temperatures on the hottest days of summer 2014. ZAMG (blue-red and dark grey areas) and ECA&D (yellow area) daily maxima and minima. The dotted black line shows calculated hourly data (ZAMG, INCA_L), the red line shows our measured temperature at 10 min intervals (M00, weather station on-site). Temperatures measured at the sample site are represented by colored lines (green: vine trellis; brown: linden tree; for details on sensors, see Table 2). CT_max_ from [39].

During the warmest summer days of 2014, the monthly means continually underestimated the temperatures in the habitat, while calculated daily data from ZAMG and ECA&D (Figure 6, blue-red and yellow areas) fitted our measurement station data (M00, red line) well. Still, temperature measurements in the areas where *P. apterus* individuals occurred regularly showed deviating values. The calculated temperature values always remained well under the CT_max_ determined for this *P. apterus* population, as did the temperatures at most points where our sensors were placed. Exceptionally high temperatures were measured when the thermocouples positioned out of shading obstacles (e.g., linden tree trunk, trellis post) were hit directly by solar radiation. However, these temperature events were limited to a few hours of the day around noon, with the sun almost vertical (Figure 6, M02, M05; sensors placed on the south side of the trunk or trellis post).

## 4. Discussion

Determining the ambient temperature as the main parameter responsible for the occurrence and spread of ectothermic insects is of paramount importance. Calculation of thermal tolerance, energetics, and distribution models depend on temperature as a driver for physiological processes underlying thriving and propagation [7,8,9]. Deviations between calculated and actual ambient temperatures at a place where organisms are present can lead to large errors in the estimation of the energy requirement. For example, Kovac et al. [48] showed that the error in calculating a basal metabolic rate (standard metabolism) or a mixed mean of resting and active metabolism of paper wasps can amount to up to 30%.

In ecophysiological research, a size ratio of 10,000:1 of area: organism size can easily occur in terms of climate parameter measurement [20]. In recent years, the importance of the integration of small-scale microclimatic data in all sorts of ecophysiological research is shown by an increasing number of scientific publications [20,22,24,49,50,51,52,53,54,55,56,57,58,59]. The value of small-scale, microclimate data—be they measured or calculated—also receives more attention in other fields such as, e.g., urban and spatial planning [60]. The World Meteorological Organization (WMO) states the E-OBS gridded dataset used in this study and the station dataset underlying it to be “*the backbone of the Climate Data node for WMO, based on a dense set of surface observations sourced directly from the National Meteorological Services*” [61]. However, the inhomogeneous positioning in space (“*with areas particularly in Europe’s south east and northern Africa poorly represented*” [61]) and time (“*with a steep increase in the number of used stations in the early 1950s*” [61]) is considered the greatest weakness of this dataset. On the European Union’s Earth observation program homepage, an entire chapter is dedicated to the known issues of the data provided on the E-OBS data store [62]. However, who would refuse the provided data if they were all one would be able to procure?

During the warmest summer days of 2014, the monthly means continually underestimated temperatures in the habitat, while calculated daily data fitted our measurement station data (M00, WSlocal) better (Figure 6). Still, temperature measurements in the areas where *P. apterus* individuals regularly occurred showed deviating values. Especially when the thermocouples positioned outside of shading obstacles were hit directly by solar radiation, exceptionally high temperatures (>50 °C) were measured (see [56] on possible shortcomings of our measurement setup). However, these temperature events were limited to a few regions during a few hours of the day (around noon, with the sun almost vertical), and *P. apterus* individuals, which are all over the place in summer, simply avoided spots with the hottest microhabitat temperatures (behavioral thermoregulation, see also [30,63]).

*Pyrrhocoris apterus* adults are not freeze-tolerant but rely on supercooling for winter survival [64,65]. Therefore, minimum ambient temperatures are critical for survival if and when they fall under the insects’ tolerable limit! In the meteorological winter (1.12.–1.03.) of 2013–14 and 2015–16, the deviation in reported ambient temperatures of the ZAMG Gleisdorf data to the local weather station at our study site was only 0.1 °C and therefore, in all likelihood, inconsequential for *P. apterus*. Only in 2014–15, ZAMG Gleisdorf differed from our weather station on site by −3.5 °C; to a degree that indeed matters for the insects’ survival (ZAMG: −13.0 °C to WSlocal: −9.5 °C; *P. apterus* CT_min_ = −4.0 °C). Monthly averages of processed gridded data, as well as our measured data, never fell below the CT_min_ of *P. apterus*. This may lead to erroneous conclusions regarding the viability of the habitat for the species because absolute temperatures exceeding or falling below thermal limits directly affect animal survival.

However, the minimum temperatures measured at the firebugs’ winter hibernaculum (M22) were always substantially higher than the air temperatures measured by ZAMG Gleisdorf (2013–14: −5.9 °C; 2014–15: −6.7 °C; 2015–16: −6.5 °C) and also our local weather station (M00; see Table 3). Our analyses show the main shortcomings of temperature data interpolated from large-scale, homogenized data (e.g., from ECA&D and ZAMG) versus data measured directly on site and in the microhabitat of the assessed insects: Large-scale estimations resulted in evidently lethal temperatures for *P. apterus*, far below the insect’s lower thermal limit (CT_min_), while actual measurements at the microhabitat in the winter hibernaculum (e.g., M22 in the cracks between cobblestones; see Figure 1, Figure 2 and Figure 3) remained above that limit most of the time, falling only occasionally below this threshold. Why the bugs chose this place as their winter hibernaculum could be explained by the following: (1) the depth limits in the positioning of the thermal sensors in the crevices between the cobblestones might have led to lower temperature readings than the majority of the bugs deeper in the crevices were exposed to (i.e., we were only able to route the sensor wire around sharp bends to a certain depth), and (2) our experiments showed that adult individuals survived supercooling at −5 °C for at least 5 min and fully regained mobility after being warmed to 15 °C afterward [39]. A change in the supercooling point throughout the seasons as winter approaches benefits the insects and will likely improve their chances of successful overwintering in our latitudes [65,66,67]. We could observe *P. apterus* individuals near their winter hibernacula in the open in early spring during the day, catching sun (see [63,64]), but also on warmer winter days (31.12.2012, measured ambient air temperature T_a_ = 3.7 °C; 23.11.2013, T_a_ = 4.3 °C, M00; our own observations). However, deceased individuals could also be found near the “entrance” to their hibernaculum (our own observations). Of course, it was impossible for us to clarify whether the individuals had died from either frostbite or other causes.

## 5. Conclusions

Temperature is a crucial physiological factor in ectothermic organisms like *Pyrrhocoris apterus*. The viable thermal niche is determined by ambient temperatures. They are lethal after only a brief exposure if the respective critical thermal maxima or minima of the insects are exceeded. Accurate determination of ambient temperatures can determine the accuracy of models or predictions of species survival and dispersal. Small-scale measurements of this parameter in an organism’s (micro)habitat are a must in order to create a durable and useful basis for models of energy requirements, survival in a changing environment, and future dispersal.

## Figures and Tables

**Table 1:** Temperature data are freely available from a selection of national and international online sources (see also Section 2.2). ZAMG is now part of GeoSphere Austria.

Dataset	Source	Data Processing
SPARTACUS	ZAMG	Gridded daily dataset of observed air temperature (°C, min, max) in 1 km resolution over Austria since 1961. Monthly and seasonal (meteorological seasons) aggregates from daily data.
INCA_L	ZAMG	Gridded background field-corrected with observational data; uses station observations, remote sensing data, numerical weather forecast models and a high-resolution terrain model; mean temperatures (°C) at 1 km × 1 km and 1 h resolution.
WS Gleisdorf	ZAMG	Monthly and daily mean, min and max temperature values (°C) from one of 260 measurement stations in Austria.
E-OBS	ECA&D	Ensemble dataset on a 0.25-degree regular grid; daily mean, min, max temperatures (°C).

**Table 3:** Deviation of minimum (winter) and maximum (summer) temperatures of processed gridded (ZAMG, E-OBS) data, as well as measured *P. apterus*’ microhabitat temperatures, from the temperatures measured with our local standardized weather station (local, M00, bold). Positive values indicate a deviation upward, and negative values indicate a deviation downward in the respective calculated or measured values in relation to our local M00 weather station. Temperatures are in °C.

		Winter, min. Temp.			Summer, Max. Temp.		
Data Source	Data Scale, Type	2013–14	2014–15	2015–16	2014	2015	2016
**M00, WS_local_**	local, measured	**−7.4**	**−9.5**	**−11.4**	**32.3**	**34.2**	**30.4**
ZAMG Gleisdorf	macro, measured	*−0.1*	*−3.5*	*−0.1*	*1.3*	*0.3*	*0.9*
ZAMG SPARTACUS	large-scale, gridded	*−0.6*	*−3.8*	*0.4*	*0.3*	*0.0*	*−0.1*
ECA&D E-OBS	large-scale, gridded	*2.8*	*3.8*	*4.2*	*0.3*	*0.1*	*0.7*
M22 Porch, hiber	micro, measured	***1.5***	***2.8***	***4.9***	***5.1***	***2.7***	***2.7***
M02 Tilia, roots, hiber	**“**	***0.8***	***3.8***	***0.9***	***7.0***	***7.1***	***4.3***
M03 Tilia trunk, north	“	*0.5*	*0.0*	*−0.4*	*1.1*	*1.1*	*0.5*
M04 Tilia crown	“	*−0.1*	*−0.5*	*−0.4*	*−0.1*	*0.6*	*−0.4*
M05 Post, south	“	*4.7*	*7.3*	*1.1*	*−6.3*	*0.1*	*−1.0*
M06 Post, north	“	*0.7*	*0.4*	*0.0*	*−3.0*	*−2.6*	*1.7*
M07 Post, soil, north, hiber	**“**	***2.7***	***6.2***	***3.4***	*−9.0*	*−3.7*	*−6.6*
M08 Post, south	“	*4.7*	*7.3*	*1.1*	*−6.3*	*0.1*	*−1.0*

**Table 4:** Averaged monthly temperatures of processed gridded data (ZAMG, E-OBS), as well as our local standardized weather station (local, M00), and measured *P. apterus*’ microhabitat temperatures of the year 2015. Temperatures are in °C; for details on the data source (location) and type (measured/gridded = provided), see Table 3.

2015												
Data Source	Jan	Feb	Mar	Apr	May	Jun	Jul	Aug	Sept	Oct	Nov	Dec
M00, WS_local_	2.0	1.2	5.2	9.7	14.4	18.3	21.5	21.0	14.3	8.9	7.0	2.4
ZAMG Gleisdorf	1.4	0.9	5.0	9.6	14.9	18.9	22.1	20.9	14.3	8.9	5.6	1.7
ZAMG SPARTACUS	1.3	1.0	5.5	9.6	14.6	18.2	21.6	20.9	14.6	9.4	6.7	1.8
ECA&D E-OBS	2.0	1.7	6.0	10.4	15.0	18.7	21.9	21.3	14.8	9.8	7.4	2.8
M22	2.9	2.9	7.5	12.3	16.4	20.9	23.6	23.8	16.4	10.6	8.6	3.8
M02	1.1	0.9	4.7	9.2	13.7	18.0	21.2	20.5	14.5	9.4	5.9	1.3
M03	1.8	1.2	5.5	10.1	14.7	18.6	21.5	21.1	14.5	9.1	7.0	2.1
M04	2.0	1.3	5.7	10.4	14.7	18.6	21.4	20.9	14.3	8.9	7.0	2.4
M05	1.3	1.0	5.9	11.4	16.1	21.4	22.9	21.9	15.1	9.7	5.9	1.5
M06	1.4	1.1	5.7	9.7	14.3	17.6	20.0	19.3	13.7	8.9	5.8	1.3
M07	1.6	1.3	4.6	9.5	14.4	17.6	19.9	19.2	13.8	9.4	6.1	2.1
M08	1.8	1.5	5.5	10.8	14.3	18.0	20.7	19.9	14.5	9.7	6.6	1.9

## Data Availability

The research was conducted using freely available temperature data from the European Climate Assessment & Dataset project (www.ecad.eu, E-OBS gridded dataset; accessed on 5 September 2022), GeoSphere Austria (https://data.hub.geosphere.at, SPARTACUS, INCA_L, station data; accessed on 27 July 2023) and our own temperature data, measured locally on site (raw data table in the Supplementary Materials).

## Supplementary Materials

The following supporting information can be downloaded at: https://www.mdpi.com/article/10.3390/insects14110843/s1, Table S1: Small-scale temperature data 2013–2016; Table S2: 2015_day_week_month.

## References

[1] Socha R. (1993). *Pyrrhocoris apterus* (Heteroptera)—An experimental model species: A review. Eur. J. Entomol..

[2] Stichel W. (1955). Illustrierte Bestimmungstabellen der Wanzen: II. Europa (Hemiptera-Heteroptera Europae).

[3] Kulik S.A. (1973). Four new species of Miridae (Heteroptera) from the Far East of the USSR. Nauchnye Dokl. Vyss. Shkoly Biol. Nauk..

[4] Marren P. (2010). Bugs Britannica.

[5] Endrestøl A., Roth S. (2020). The firebug *Pyrrhocoris apterus* (Linnaeus, 1758) (Hemiptera, Heteroptera) new to the Norwegian fauna—With an explosive expansion in Northern Europe. Nor. J. Entomol..

[6] Mata L., Vogel B., Palma E., Malipatil M. (2022). The Arrival and Spread of the European Firebug (Pyrrhocoris apterus) in Australia as Documented by Citizen Scientists. Urban Nat. Notes.

[7] Taylor F. (1981). Ecology and Evolution of Physiological Time in Insects. Am. Nat..

[8] Chown S.L., Nicolson S.W. (2004). Insect Physiological Ecology: Mechanisms and Patterns.

[9] Dixon A.F., Honěk A., Keil P., Kotela M.A.A., Šizling A.L., Jarošík V. (2009). Relationship between the minimum and maximum temperature thresholds for development in insects. Funct. Ecol..

[10] Bale J.S. (1991). Insects at Low Temperature: A Predictable Relationship?. Funct. Ecol..

[11] Sunday J.M., Bates A.E., Dulvy N.K. (2011). Global analysis of thermal tolerance and latitude in ectotherms. Proc. Biol. Sci..

[12] Addo-Bediako A., Chown S.L., Gaston K.J. (2000). Thermal tolerance, climatic variability and latitude. Proc. Biol. Sci..

[13] Sunday J.M., Bates A.E., Kearney M.R., Colwell R.K., Dulvy N.K., Longino J.T., Huey R.B. (2014). Thermal-safety margins and the necessity of thermoregulatory behavior across latitude and elevation. Proc. Natl. Acad. Sci. USA.

[14] Winwood-Smith H.S., Alton L.A., Franklin C.E., White C.R. (2015). Does greater thermal plasticity facilitate range expansion of an invasive terrestrial anuran into higher latitudes?. Conserv. Physiol..

[15] Ducatez S., Baguette M., Trochet A., Chaput-Bardy A., Legrand D., Stevens V., Fréville H. (2013). Flight endurance and heating rate vary with both latitude and habitat connectivity in a butterfly species. Oikos.

[16] Alford L., Blackburn T.M., Bale J.S. (2012). Effect of latitude and acclimation on the lethal temperatures of the peach-potato aphid Myzus persicae. Agric. For. Entomol..

[17] Buckley L.B., Huey R.B. (2016). Temperature extremes: Geographic patterns, recent changes, and implications for organismal vulnerabilities. Glob. Chang. Biol..

[18] Deutsch C.A., Tewksbury J.J., Huey R.B., Sheldon K.S., Ghalambor C.K., Haak D.C., Martin P.R. (2008). Impacts of climate warming on terrestrial ectotherms across latitude. Proc. Natl. Acad. Sci. USA.

[19] Hoffmann A.A. (2010). Physiological climatic limits in *Drosophila*: Patterns and implications. J. Exp. Biol..

[20] Potter K.A., Arthur Woods H., Pincebourde S. (2013). Microclimatic challenges in global change biology. Glob. Chang. Biol..

[21] Rebaudo F., Faye E., Dangles O. (2016). Microclimate Data Improve Predictions of Insect Abundance Models Based on Calibrated Spatiotemporal Temperatures. Front. Physiol..

[22] Pincebourde S., Casas J. (2015). Warming tolerance across insect ontogeny: Influence of joint shifts in microclimates and thermal limits. Ecology.

[23] Kovac H., Käfer H., Petrocelli I., Stabentheiner A. (2022). The respiratory metabolism of overwintering paper wasp gynes (*Polistes dominula* and *Polistes gallicus*). Physiol. Entomol..

[24] Pincebourde S., Salle A. (2020). On the importance of getting fine-scale temperature records near any surface. Glob. Chang. Biol..

[25] MacLean H.J., Sørensen J.G., Kristensen T.N., Loeschcke V., Beedholm K., Kellermann V., Overgaard J. (2019). Evolution and plasticity of thermal performance: An analysis of variation in thermal tolerance and fitness in 22 *Drosophila* species. Philos. Trans. R. Soc. B Biol. Sci..

[26] Hadley N.F. (1970). Water Relations of The Desert Scorpion, *Hadrurus arizonensis*. J. Exp. Biol..

[27] Hadley N.F., Quinlan M.C., Kennedy M.L. (1991). Evaporative cooling in the desert cicada. J. Exp. Biol..

[28] Buckley L.B., Miller E.F., Kingsolver J.G. (2013). Ectotherm thermal stress and specialization across altitude and latitude. Integr. Comp. Biol..

[29] Kearney M.R., Shine R., Porter W.P. (2009). The potential for behavioral thermoregulation to buffer “cold-blooded” animals against climate warming. Proc. Natl. Acad. Sci. USA.

[30] Woods H.A., Dillon M.E., Pincebourde S. (2015). The roles of microclimatic diversity and of behavior in mediating the responses of ectotherms to climate change. J. Therm. Biol..

[31] ZAMG—Zentralanstalt für Meteorologie und Geodynamik ZAMG Data Hub. https://data.hub.zamg.ac.at.

[32] UERRA—Uncertainities in Ensembles of Regional Analyses E-OBS Dataset from the EU-FP6 Project UERRA. https://www.uerra.eu/.

[33] Copernicus—Europe’s Eyes on Earth E-OBS Daily Gridded Meteorological Data for Europe from 1950 to Present Derived from In-Situ Observations. https://cds.climate.copernicus.eu/cdsapp#!/dataset/insitu-gridded-observations-europe?tab=overview.

[34] Cornes R.C., van der Schrier G., van den Besselaar E.J.M., Jones P.D. (2018). An Ensemble Version of the E-OBS Temperature and Precipitation Data Sets. J. Geophys. Res. Atmos..

[35] Beck H.E., Zimmermann N.E., McVicar T.R., Vergopolan N., Berg A., Wood E.F. (2018). Present and future Köppen-Geiger climate classification maps at 1-km resolution. Sci. Data.

[36] Kottek M., Grieser J., Beck C., Rudolf B., Rubel F. (2006). World Map of the Köppen-Geiger climate classification updated. Metz.

[37] Peel M.C., Finlayson B.L., McMahon T.A. (2007). Updated world map of the Köppen-Geiger climate classification. Hydrol. Earth Syst. Sci..

[38] Käfer H., Kovac H., Stabentheiner A., Simov N., Battisti A. (2018). Thermal traits of true bugs, an insect taxon with high invasive potential. ECE 2018—XI European Congress of Entomology, Book of Abstracts, Proceedings of the XI European Congress of Entomology, Naples, Italy, 2–6 July 2018.

[39] Käfer H., Kovac H., Simov N., Battisti A., Erregger B., Schmidt A.K.D., Stabentheiner A. (2020). Temperature Tolerance and Thermal Environment of European Seed Bugs. Insects.

[40] Andersen J.L., Manenti T., Sørensen J.G., MacMillan H.A., Loeschcke V., Overgaard J. (2015). How to assess *Drosophila* cold tolerance: Chill coma temperature and lower lethal temperature are the best predictors of cold distribution limits. Funct. Ecol..

[41] Chown S.L., Jumbam K.R., Sørensen J.G., Terblanche J.S. (2009). Phenotypic variance, plasticity and heritability estimates of critical thermal limits depend on methodological context. Funct. Ecol..

[42] Terblanche J.S., Deere J.A., Clusella-Trullas S., Janion C., Chown S.L. (2007). Critical thermal limits depend on methodological context. Proc. R. Soc. Lond. B.

[43] Kovac H., Käfer H., Stabentheiner A. (2020). The Respiratory Metabolism of *Polistes biglumis*, a Paper Wasp from Mountainous Regions. Insects.

[44] Lutterschmidt W.I., Hutchison V.H. (1997). The critical thermal maximum: Data to support the onset of spasms as the definitive end point. Can. J. Zool..

[45] Hazell S.P., Pedersen B.P., Worland M.R., Blackburn T.M., Bale J.S. (2008). A method for the rapid measurement of thermal tolerance traits in studies of small insects. Physiol. Entomol..

[46] Klok C.J., Chown S.L. (1997). Critical thermal limits, temperature tolerance and water balance of a sub-Antarctic caterpillar, *Pringleophaga marioni* (Lepidoptera: Tineidae). J. Insect Physiol..

[47] Pinsky M.L., Eikeset A.M., McCauley D.J., Payne J.L., Sunday J.M. (2019). Greater vulnerability to warming of marine versus terrestrial ectotherms. Nature.

[48] Kovac H., Käfer H., Petrocelli I., Amstrup A.B., Stabentheiner A. (2022). Energetics of Paper Wasps (*Polistes* sp.) from Differing Climates during the Breeding Season. Insects.

[49] Pincebourde S., Suppo C. (2016). The Vulnerability of Tropical Ectotherms to Warming Is Modulated by the Microclimatic Heterogeneity. Integr. Comp. Biol..

[50] Pincebourde S., Woods H.A. (2020). There is plenty of room at the bottom: Microclimates drive insect vulnerability to climate change. Curr. Opin. Insect Sci..

[51] Kearney M.R., Isaac A.P., Porter W.P. (2014). microclim: Global estimates of hourly microclimate based on long-term monthly climate averages. Sci. Data.

[52] Kearney M.R., Shamakhy A., Tingley R., Karoly D.J., Hoffmann A.A., Briggs P.R., Porter W.P., Travis J. (2014). Microclimate modelling at macro scales: A test of a general microclimate model integrated with gridded continental-scale soil and weather data. Methods Ecol. Evol..

[53] Kearney M.R., Gillingham P.K., Bramer I., Duffy J.P., Maclean I.M. (2020). A method for computing hourly, historical, terrain-corrected microclimate anywhere on earth. Methods Ecol. Evol..

[54] Kearney M.R., Porter W.P. (2009). Mechanistic niche modelling: Combining physiological and spatial data to predict species’ ranges. Ecol. Lett..

[55] Porter W.P., Ostrowski S., Williams J.B. (2010). Modeling Animal Landscapes. PBZ.

[56] Maclean I.M.D., Duffy J.P., Haesen S., Govaert S., de Frenne P., Vanneste T., Lenoir J., Lembrechts J.J., Rhodes M.W., van Meerbeek K. (2021). On the measurement of microclimate. Methods Ecol. Evol..

[57] Maclean I.M.D. (2019). Predicting future climate at high spatial and temporal resolution. Glob. Chang. Biol..

[58] Lembrechts J.J., Nijs I., Lenoir J. (2019). Incorporating microclimate into species distribution models. Ecography.

[59] Nadeau C.P., Urban M.C., Bridle J.R. (2017). Coarse climate change projections for species living in a fine-scaled world. Glob. Chang. Biol..

[60] Eingrüber N., Korres W., Schneider K. (2022). Microclimatic field measurements to support microclimatological modelling with ENVI-met for an urban study area in Cologne. Adv. Sci. Res..

[61] World Meteorological Organisation Climate Observation Networks. https://community.wmo.int/climate-observation-networks.

[62] Copernicus—Europe’s Eyes on Earth Known Issues in E-OBS. https://surfobs.climate.copernicus.eu/userguidance/known_issues_eobs.php.

[63] Honek A., Martinkova Z. (2019). Behavioural thermoregulation hastens spring mating activity in *Pyrrhocoris apterus* (Heteroptera: Pyrrhocoridae). J. Therm. Biol..

[64] Koštál V., Šimek P. (2000). Overwintering strategy in *Pyrrhocoris apterus* (Heteroptera): The relations between life-cycle, chill tolerance and physiological adjustments. J. Insect Physiol..

[65] Hodkova M., Hodek I. (1997). Temperature Regulation of Supercooling and Gut Nucleation in Relation to Diapause of *Pyrrhocoris apterus* (L.) (Heteroptera). Cryobiology.

[66] Ditrich T. (2018). Supercooling point is an individually fixed metric of cold tolerance in *Pyrrhocoris apterus*. J. Therm. Biol..

[67] Ditrich T., Janda V., Vaněčková H., Doležel D. (2018). Climatic Variation of Supercooling Point in the Linden Bug *Pyrrhocoris apterus* (Heteroptera: Pyrrhocoridae). Insects.

